# Rg3-enriched ginseng extract ameliorates scopolamine-induced learning deficits in mice

**DOI:** 10.1186/s12906-016-1050-z

**Published:** 2016-02-18

**Authors:** Jiyoung Kim, Jaesung Shim, Siyoung Lee, Woo-Hyun Cho, Eunyoung Hong, Jin Hee Lee, Jung-Soo Han, Hyong Joo Lee, Ki Won Lee

**Affiliations:** WCU Biomodulation Major, Department of Agricultural Biotechnology, Seoul National University, Seoul, 151-742 Republic of Korea; Center for Food and Bioconvergence, Seoul National University, Seoul, 151-921 Republic of Korea; Research Institute for Veterinary Science, College of Veterinary Medicine, Seoul National University, Seoul, 151-742 Republic of Korea; Department of Biological Sciences, Konkuk University, Seoul, 143-701 Republic of Korea; Foods R&D, CJ Cheiljedang Corp., Seoul, 152-051 Republic of Korea; Department of Food Science and Biotechnology, CHA University, Pocheon, Gyeonggi-do 487-010 Republic of Korea; Advanced Institutes of Convergence Technology, Seoul National University, Suwon, 443-270 Republic of Korea; Research Institute of Bio Food Industry, Institute of Green Bio Science and Technology, Seoul National University, Pyeongchang, 232-916 Republic of Korea

**Keywords:** **G**inseng, Rg3, Scopolamine, Memory, Acetylcholinesterase, NF-κB

## Abstract

**Background:**

Ginseng (*Panax ginseng* C.A. Meyer) has been used as a traditional herb in the treatment of many medical disorders. Ginsenosides, which are triterpene derivatives that contain sugar moieties, are the main pharmacological ingredients in ginseng. This study was designed to investigate the effect of ginsenoside Rg3-enriched ginseng extract (Rg3GE) on scopolamine-induced memory impairment in mice.

**Methods:**

Rg3GE (50 and 100 mg/kg) were administered to C57BL/6 mice by oral gavage for 14 days (days 1–14). Memory impairment was induced by scopolamine (1 mg/kg, intraperitoneal injection) for 6 days (days 914). The Morris water maze test was used to assess hippocampus-dependent spatial memory. The effects of scopolamine with or without Rg3GE on acetylcholinesterase and nuclear factor-κB (NF-κB) in the hippocampus were also examined.

**Results:**

Mice with scopolamine treatment alone showed impairments in the acquisition and retention of spatial memory. Mice that received Rg3GE and scopolamine showed no scopolamine-induced impairment in the acquisition of spatial memory. Oral administration of Rg3GE suppressed the scopolamine-mediated increase in acetylcholinesterase activity and stimulation of the NF-κB pathway (i.e., phosphorylation of p65) in the hippocampus.

**Conclusion:**

These findings suggest that Rg3GE may stabilize scopolamine-induced memory deficits through the inhibition of acetylcholinesterase activity and NF-κB signaling in the hippocampus.

## Background

**Fig. 1:**
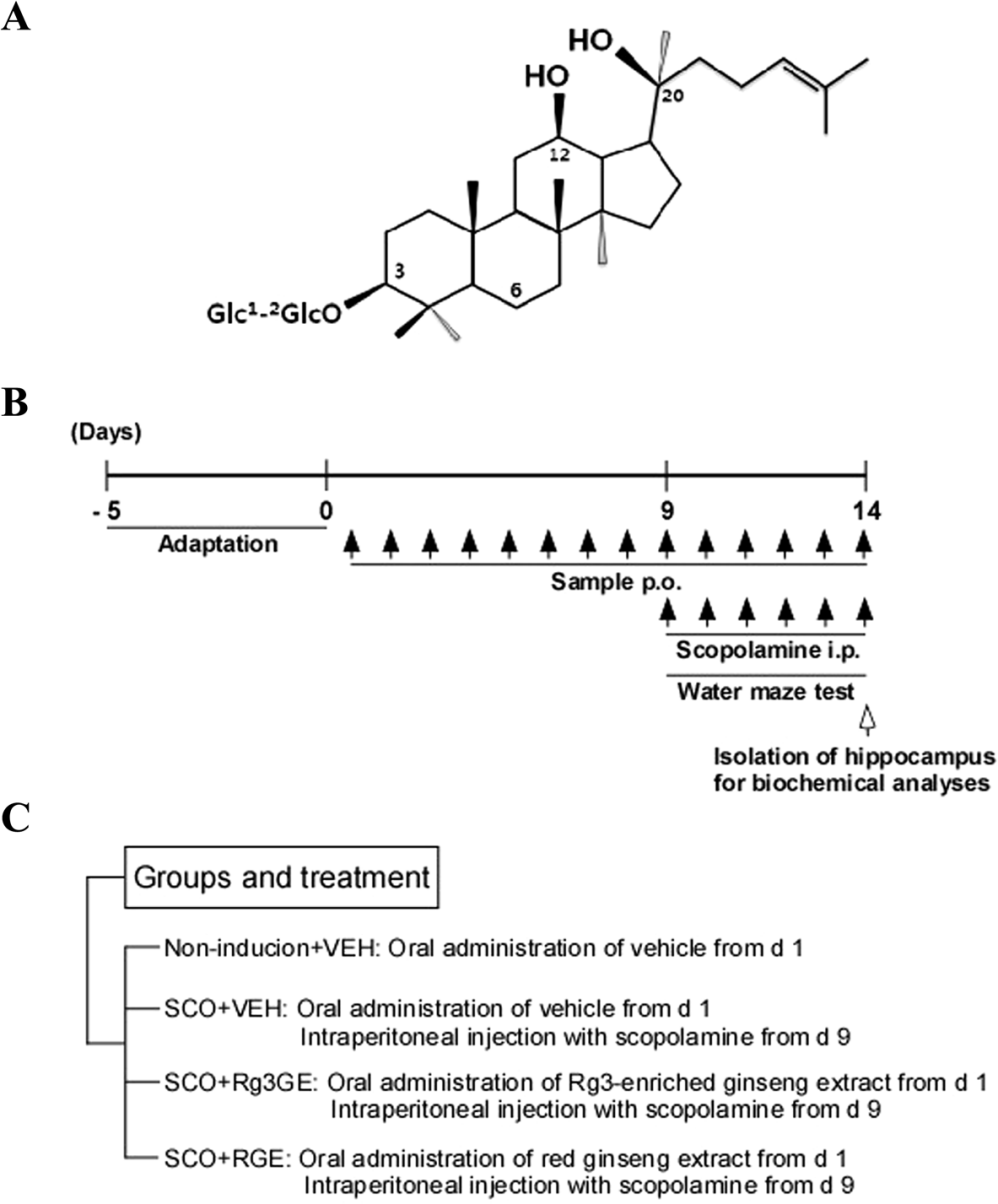
Chemical structure of Rg3 and study design. **a** Structure of Rg3. **b** After a 5-day adaption period, the mice were given Rg3GE (50 or 100 mg/kg, p.o.) or RGE (100 mg/kg, p.o.) for a total of 14 days. Beginning on day 9, scopolamine (1 mg/kg, i.p.) was also administered for 6 days, and the Morris water maze test was used to assess behavior during this period (days 9–14). On day 14, each mouse was sacrificed, and the hippocampus was removed for the analysis of acetylcholinesterase activity and NF-κB activation. **c** Groups and treatment

Ginseng, the dried root of *Panax ginseng* C. A. Meyer, has long been used as a traditional medicine in Asian countries [1]. It is commonly used for enhancing body strength, recovering physical balance, and stimulating metabolic function [1]. Many active ingredients of *Panax ginseng*, including ginsenosides, ginseng peptides, and ginseng polysaccharides, have been studied extensively for their medicinal properties [2, 3]. Among them, 20(*S*)-ginsenoside Rg3, which consists of three parts: a steroid-like backbone structure, a carbohydrate portion, and an aliphatic side chain (−CH_2_CH_2_CH = C(CH_3_)_2_) coupled to carbon-20 of the backbone structure (Fig. 1a), exhibits neuroprotective effects in cultured cells and experimental animals [4–6].

The central cholinergic system plays an important role in regulating memory function. The neurotransmitter acetylcholine controls numerous cognitive processes [7]. Scopolamine is a nonselective muscarinic acetylcholine receptor antagonist that interferes with the processes of learning acquisition and short-term memory in animals and humans [8, 9]. The hippocampus is particularly vulnerable to scopolamine-induced neuronal injury [10]. Scopolamine increases acetylcholinesterase activity in the hippocampus [11]. The scopolamine-induced memory impairment model is non-degenerative and shows cholinergic dysregulation for which no other relevant models exist. Thus, scopolamine has been used to generate experimental animal models for the screening of anti-amnesic drugs [12].

Ginsenoside Rg3 is not naturally present in *Panax ginseng* [13] and is generated by the loss of the glucose group linked with the C-20 of ginsenoside Rc, Rd, Rb1, or Rb2 when ginseng roots are heated to a high temperature [14]. It has been reported that red ginseng, which is prepared by steaming ginseng root at 98–100 °C, only contains ginsenoside Rg3 [15]. Since ginsenoside Rg3 does not naturally occur in *Panax ginseng* [13], we prepared ginseng extract (GE), which is enriched in Rg3 (14.34 ± 0.28 mg/g of Rg3s and Rg3r (Rg3[s + r])) to investigate whether Rg3GE has an ameliorating effect on learning and memory impairment. The Morris water maze test was used to assess its effect on the scopolamine-induced impairment of hippocampus-dependent spatial memory. We also examined the effects of Rg3GE on scopolamine-induced acetylcholinesterase activity and nuclear factor (NF)-κB signaling in the hippocampus.

## Methods

### Materials

Red ginseng extract (RGE), which contains 2.63 ± 0.02 mg/g of Rg3 (s + r), was purchased from KT&G (Daejeon, South Korea) and used as a positive control. Scopolamine hydrochloride was purchased from Sigma-Aldrich (St. Louis, MO, USA). The Amplex Red Acetylcholine/Acetylcholinesterase assay kit was purchased from Invitrogen (Grand Island, NY, USA). Antibodies against phosphorylated p65 (p-p65) and β-actin were purchased from Cell Signaling Technology (Danvers, MA, USA) and Sigma-Aldrich, respectively.

### Preparation of Rg3GE

Rg3-enriched ginseng extract (Rg3GE) was prepared from the roots of 4-year-old dried Korean ginseng (*Panax ginseng* C.A. Meyer) from Ginseng Nonghyup (Punggi, Korea). The ginseng root was extracted three times with 10 volumes of 70 % fermentation ethanol by heat-reflux extraction at 70 °C for 4 h, and then concentrated twice under vacuum at 60 °C and 600–700 mmHg. After extracting in 10 volumes of distilled water at 100 °C for 3 h, the extract was filtered and evaporated. Finally, the extract solution was lyophilized to obtain powdered ginseng extract rich in ginsenoside Rg3 (Rg3GE).

**Fig. 2:**
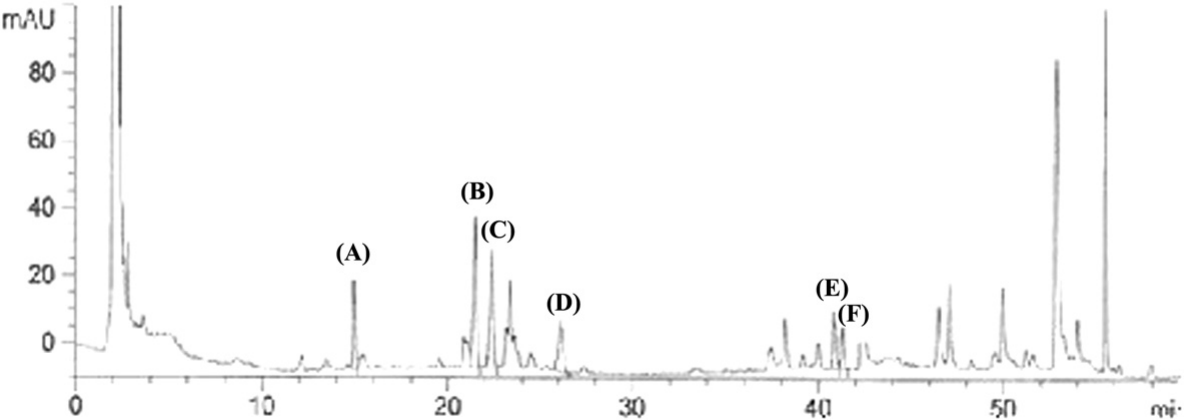
High-performance liquid chromatography pattern of Rg3GE used in this study. Rg3(s + r) (14.34 ± 0.28 mg/g) was found in Rg3GE. Peaks: (A) Rg1, (B) Rb1, (C) Rc, (D) Rd, (E) Rg3(s), (F) Rg3(r)

Ginsenoside levels were quantitatively determined using a high-performance liquid chromatography (HPLC) system (Agilent, Palo Alto, CA, USA), consisting of an Agilent 1260 Infinity Quaternary Pump (G1311B), standard auto-sampler (G1329B), and thermostat column compartment (G1316B) equipped with a Peltier cooling and heating apparatus. Chromatographic separation of major ginsenosides was performed on a C18-bonded reversed-phase silica column (Venusil XBP C18, 250 × 4.6 mm, i.d. 5 μm, Bonna-Agela Technology, Newark, DE, USA) at 30 °C. All samples were filtered with a 0.45-μm membrane before being placed into chromatographic vials (Agilent), and then placed on the auto-sampler tray. The HPLC conditions were as follows: solvent A, water; solvent B, acetonitrile; gradient, 0–20 min (20–24 % B), 20–23 min (24–27 % B), 23–45 min (27–40 % B), 45–65 min (40–70 % B), 65–80 min (70–100 % B), 80–90 min (100–20 % B) with a flow rate of 1.0 ml/min and an injection volume of 20 μl. The detection was carried out at 203 nm and the total run time was 90 min. Each standard ginsenoside was injected for the HPLC analysis, and peaks were assigned by comparing the retention time (RT) of each peak with that of each reference compound. The standard ginsenosides Rg1, Rb1, Rc, and Rd were obtained from Wako Pure Chemical Industries, Ltd (Osaka, Japan). Ginsenoside Rg3(s) and Rg3(r) were from BTGin (Daejeon, Korea). HPLC analysis of Rg3GE showed that Rg3GE contains ginsenoside Rg1 (A; 16.61 ± 1.12 mg/g; RT, 14.3 min), Rb1 (B; 47.97 ± 0.59 mg/g; RT, 21.4 min), Rc (C; 39.56 ± 1.67 mg/g; RT, 22.3 min), Rd (D; 17.13 ± 0.65 mg/g; RT, 26.13 min) and Rg3(s + r) (E and F; 14.34 ± 0.28 mg/g; RT, 40.8 min and 41.3 min) (Fig. 2).

### Animals

Male C57BL/6 mice (10 weeks, 25–27 g) were purchased from NARA biotech (Seoul, South Korea) and housed in a regulated environment (temperature, 21 ± 2 °C; 12-h light/dark cycle; light period starting at 8 AM) with free access to food and water. All experimental studies were submitted to and approved by Konkuk University’s Council Directive for the Care and Use of Laboratory Animals (KU11025). To test the effect of Rg3GE on cognitive function, the mice were randomly assigned to five treatment groups (*n* = 10 per group, Fig. 1c): 1) non-induction + vehicle (deionized water), 2) vehicle + scopolamine (1 mg/kg/day, 100 μl/day), 3) Rg3GE (50 mg/kg/day, 150 μl/day) + scopolamine (1 mg/kg/day, 100 μl/day), 4) Rg3GE (100 mg/kg/day, 150 μl/day) + scopolamine (1 mg/kg/day, 100 μl/day), and 5) RGE (100 mg/kg/day, 150 μl/day) + scopolamine (1 mg/kg/day, 100 μl/day). Group 5 was the positive control. Rg3GE and RGE were suspended in deionized water and administered by oral gavage (p.o.) on days 1–8, prior to training for the Morris water maze test, and continued on days 9–14, during which the mice underwent the Morris water maze test. Scopolamine was dissolved in deionized water and administered by intraperitoneal (i.p.) injection on days 9–14. On days 9–14, Rg3GE and RGE were administered 60 min before the trial, and scopolamine was injected 30 min before the trial. Since scopolamine causes temporary cognitive deficit [16, 17], animal models for amnesia using scopolamine have been developed by injecting scopolamine 30 to 60 min before measuring memory deficit. To investigate the anti-amnesic activity of drugs, the protocol which applies test drugs before the scopolamine injection is being used [11, 18–26]. Mouse weights were measured during the treatments with Rg3GE, RGE, and scopolamine on days 1–14. On day 14, the mice were decapitated immediately following the Morris water maze test. The hippocampus was removed, dissected on ice, and frozen at −80 °C until analysis. Four or three mice hippocampi were randomly selected for acetylcholinesterase activity or western blot analysis, respectively. They were homogenized using Bullet Blender (Next Advance, Averill Park, NY, USA) in ice-cold Tissue Protein Extraction Reagent (Thermo Fisher Scientific, Rockford, IL, USA) containing Phosphatase Inhibitor Cocktail (Sigma-Aldrich), 0.1 mM phenylmethanesulfonylfluoride, and 10 μg/ml leupetin. Lysates were centrifuged at 19,000 × g for 15 min, and the protein concentrations of the supernatants were determined using the Protein Assay Reagent (Bio-Rad, Hercules, CA, USA). The experimental design is summarized in Fig. 1b.

### Morris water maze test

The water maze test was conducted in a circular tank (diameter 183 cm, height 58 cm) filled with water maintained at 25 ± 2 °C. The tank was divided into four quadrants, with a hidden escape platform (diameter, 20 cm; height, 48 cm) submerged 1.5 cm below the water surface in the center of one quadrant. The mice were trained to find the hidden platform by learning and memorizing several visual cues placed outside the maze. The position of the cues remained unchanged throughout the experiments. Four trials were conducted on each day of the training period (days 9–14). Each mouse was given 60 s to find the hidden platform and allowed to remain on it for another 30 s. The swimming speed of each mouse was measured for days 9–14 during the Morris water maze test. The mean distance from the platform (mean search error) during training trials was determined. Mice that failed to escape from the water within 60 s were guided to the platform and allowed to remain there for 30 s. During the training (acquisition) trials, search errors that are described in detail elsewhere [27] were used to assess the performance accuracy of spatial learning in the water maze. During each trial, the distance of the mice from the escape platform was sampled 10 times per second, and these values were averaged in 1-s bins. The cumulative search error was then calculated as the summed 1-s averages of the proximity measures corrected for the particular start location in each trial. On day 12 and 14, 30 min after the last trial, the platform was removed from the tank, and the mice underwent a spatial probe trial in which they were given 60 s to search for the removed platform. The time spent in the target quadrant was measured. A camera located above the center of the maze relayed images to a videocassette recorder. Data from the water maze trials were analyzed using the human visual system Image Software (HVS Image, Hampton, UK).

### Acetylcholinesterase activity assay

The Amplex Red Acetylcholine/Acetylcholinesterase assay kit was used to determine acetylcholinesterase activity. Working solution of 400 μM Amplex Red reagent containing 2 U/ml horseradish peroxidase and 0.2 U/ml choline oxidase was prepared from stock solution, and 100 μM ACh was added to measure AChE activity. The reaction began when 100 μl of the working solution was added to microplate wells containing hippocampus lysates. Fluorescence emitted by individual samples was detected using a VersaMax ELISA microplate reader (Molecular Devices, Sunnyvale, CA, USA) at an excitation wavelength of 560 nm and emission wavelength of 590 nm. Background fluorescence was eliminated by subtracting values derived from the negative control.

### Western blot analysis

The proteins (100 μg) from hippocampus lysates were separated by 10 % sodium dodecyl sulfate polyacrylamide gel electrophoresis and electrophoretically transferred to a polyvinylidene fluoride membrane (Millipore, Billerica, MA, USA). The membrane was blocked in 5 % fat-free dry milk and then incubated with primary antibodies against p-p65 and β-actin. After incubation with horseradish-peroxidase-conjugated secondary antibodies, protein bands were detected using an enhanced chemiluminescence detection kit (GE Healthcare, St. Giles, UK).

### Statistical analysis

Statistical analyses were performed using SPSS 19.0 for Windows (SPSS Inc., Chicago, IL, USA). Results were expressed as mean ± standard error of the mean (SEM). Morris water maze data were analyzed by one-way repeated analysis of variance (ANOVA), followed by Fisher’s least significant difference (LSD) test. The probe test and remaining data were analyzed by one-way ANOVA, followed by Tukey’s post-hoc test.

## Results

### Rg3GE inhibited scopolamine-induced impairment of spatial memory

**Fig. 3:**
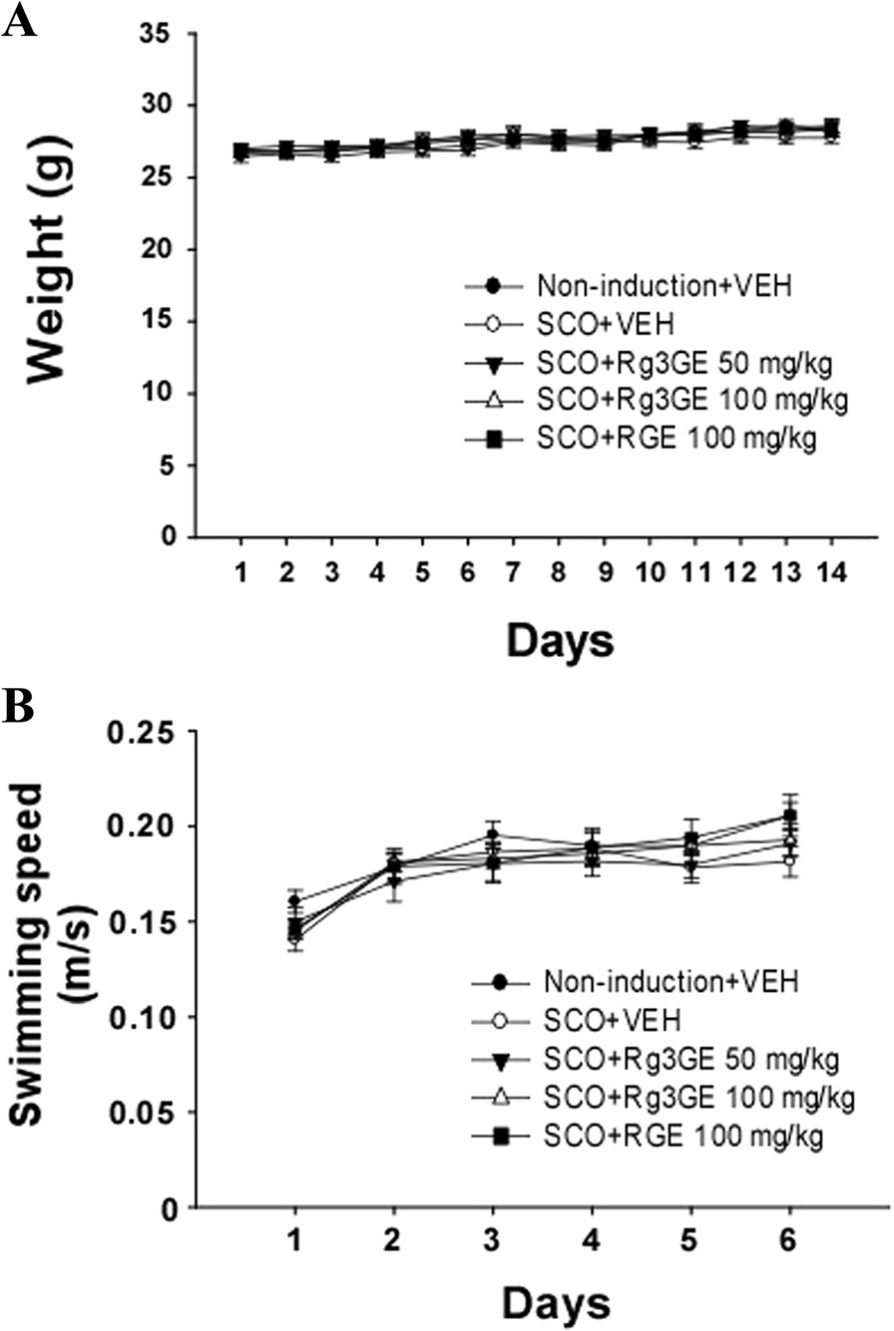
Effects of Rg3GE or RGE with scopolamine on the (**a**) weight and (**b**) speed of mice. Mouse body weights were measured on days 1–14, during treatment with Rg3GE (50 or 100 mg/kg, p.o.), RGE (100 mg/kg, p.o.), and scopolamine (1 mg/kg, i.p.). The swimming speed of each mouse was also measured for days 9–14 during the training trials of the Morris water maze test. Statistical analysis was performed by one-way repeated ANOVA followed by Fisher’s LSD test. Data are expressed as mean ± SEM (*n* = 10)

To determine the effects of Rg3GE in cognitive function, the Morris water maze test was performed. We first investigated whether Rg3GE, RGE, or scopolamine alters the body weight or swimming speed of each mouse. Mouse body weights were measured on days 1–14 during the treatments with Rg3GE, RGE, and scopolamine, and no significant differences were found between the different groups (non-induction + vehicle, scopolamine + vehicle, scopolamine + Rg3GE, scopolamine + RGE; F(4,45) = 0.662; *p* = 0.621; Fig. 3a). The swimming speed of each mouse was also measured for days 9–14 during the training trials of the Morris water maze test, and these values were also not found to be significantly different between groups (non-induction + vehicle, scopolamine + vehicle, scopolamine + Rg3GE, scopolamine + RGE; F(4,45) = 0.613; *p* = 0.656; Fig. 3b). These data suggest that Rg3GE, RGE, and scopolamine do not affect the body weight or swimming speed of the mice.

**Fig. 4:**
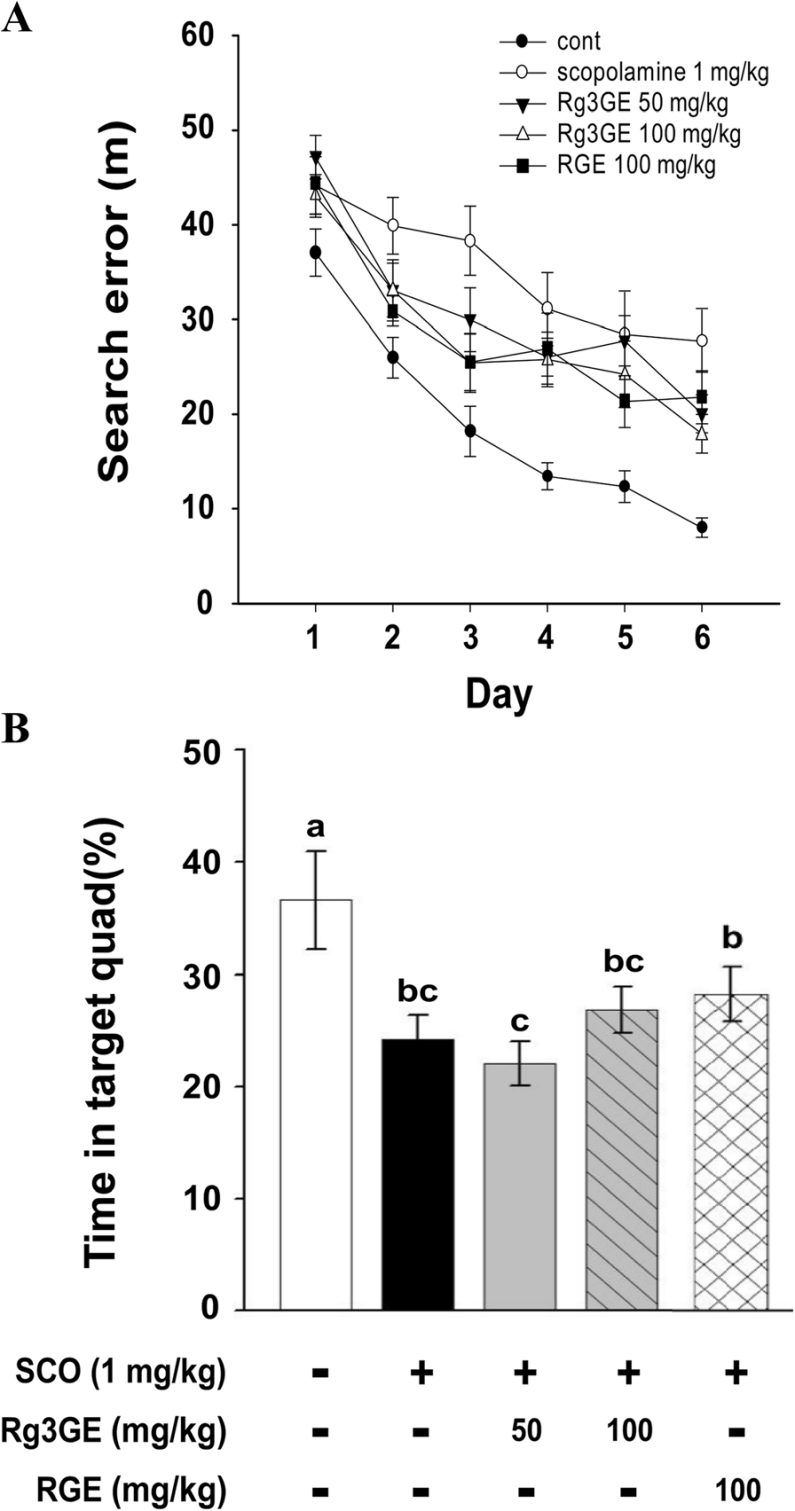
Effect of Rg3GE on scopolamine-induced impairment of spatial memory during the training period for the Morris water maze test. **a** Rg3GE (50 or 100 mg/kg, p.o.) or RGE (100 mg/kg, p.o.) was administered prior to the training trials for the Morris water maze test (days 1–8) and continued on days 9–14, during which the Morris water maze test was carried out. Impairment of spatial memory was induced by scopolamine (SCO, 1 mg/kg, administered by i.p. injection on days 9–14). During the training period (days 9–14), we evaluated the mean search error. **b** On days 12 and 14, probe trials were conducted to measure the retention of spatial memory. In the probe trial on day 14, vehicle control mice spent significantly more time in the target quadrant than did scopolamine-treated mice (#). However, Rg3GE or RGE treatments did not improve performance of the scopolamine-treated mice. Statistical analysis was performed by one-way repeated ANOVA, followed by Fisher’s LSD test. Data are expressed as mean ± SEM (*n* = 10)

Analysis of mean search error (i.e., mean distance from the escape platform during the search) revealed significant differences between the groups (non-induction + vehicle, scopolamine + vehicle, scopolamine + Rg3GE, scopolamine + RGE; F(5,54) = 8.948; *p* < 0.001), as well as with training (F(5270) = 61.579, *p* < 0.001). However, a significant interaction between treatment and training session was not detected (F(25,270) = 0.724; *p =* n.s). As shown in Fig. 4, the control mice became proficient at locating the submerged platform during the training sessions; however, scopolamine + vehicle-treated mice showed little improvement over the course of training. Post-hoc analysis confirmed that the mean search error of the control mice was significantly lower than that of scopolamine + vehicle-treated mice (*p* < 0.001). However, scopolamine-treated mice that also received Rg3GE (100 mg/kg, but not 50 mg/kg) or RGE (100 mg/kg) performed significantly better than those that received scopolamine alone (Rg3GE 50 mg/kg: *p* =0.137; Rg3GE 100 mg/kg: *p* = 0.018; RGE 100 mg/kg: *p* = 0.022; Fig. 4a). The effect of Rg3GE at 100 mg/kg on the scopolamine-induced learning deficit was similar compared to RGE at 100 mg/kg (*p* = 0.935). These observations suggest that Rg3GE inhibited the scopolamine-induced impairment of spatial memory acquisition.

On day 12, the mice underwent the spatial probe test to determine whether they remembered the platform location. One-way ANOVA revealed no significant differences between treatment groups. On day 14, the mice underwent another spatial probe test. One-way ANOVA revealed significant differences between the treatment groups (F(5, 270) = 2.664; *p* < 0.05). Subsequent post-hoc analyses revealed significant differences between the vehicle-treated and scopolamine-treated mice (*p* < 0.05; Fig. 4b). However, Rg3GE or RGE treatment did not result in any improvement in spatial memory retention.

### Rg3GE suppressed scopolamine-induced acetylcholinesterase activity

**Fig. 5:**
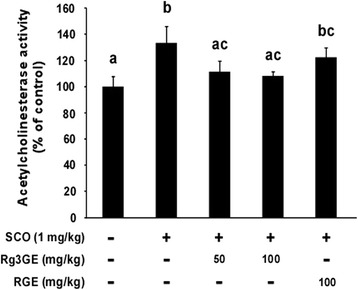
Effect of Rg3GE on scopolamine-induced acetylcholinesterase activation. Mice were decapitated immediately following the Morris water maze test, and the hippocampi were dissected to assay acetylcholinesterase activity. Statistical analysis was performed by one-way ANOVA followed by Turkey’s post-hoc test. Data are expressed as mean ± SEM (*n* = 4). Letters (a–c) within a graph are significantly different from each other at *p* < 0.05

We examined the effect of Rg3GE on acetylcholinesterase activity in the hippocampus (Fig. 5). Scopolamine + vehicle-treated mice showed significantly increased acetylcholinesterase activity compared with non-induction + vehicle-treated control mice. Rg3GE (50 and 100 mg/kg) administration significantly inhibited acetylcholinesterase activity, compared with that of the scopolamine + vehicle-treated group. Interestingly, RGE (50 mg/kg) administration did not have an effect on acetylcholinesterase activity in the hippocampus.

### Rg3GE suppressed scopolamine-induced NF-κB activation

**Fig. 6:**
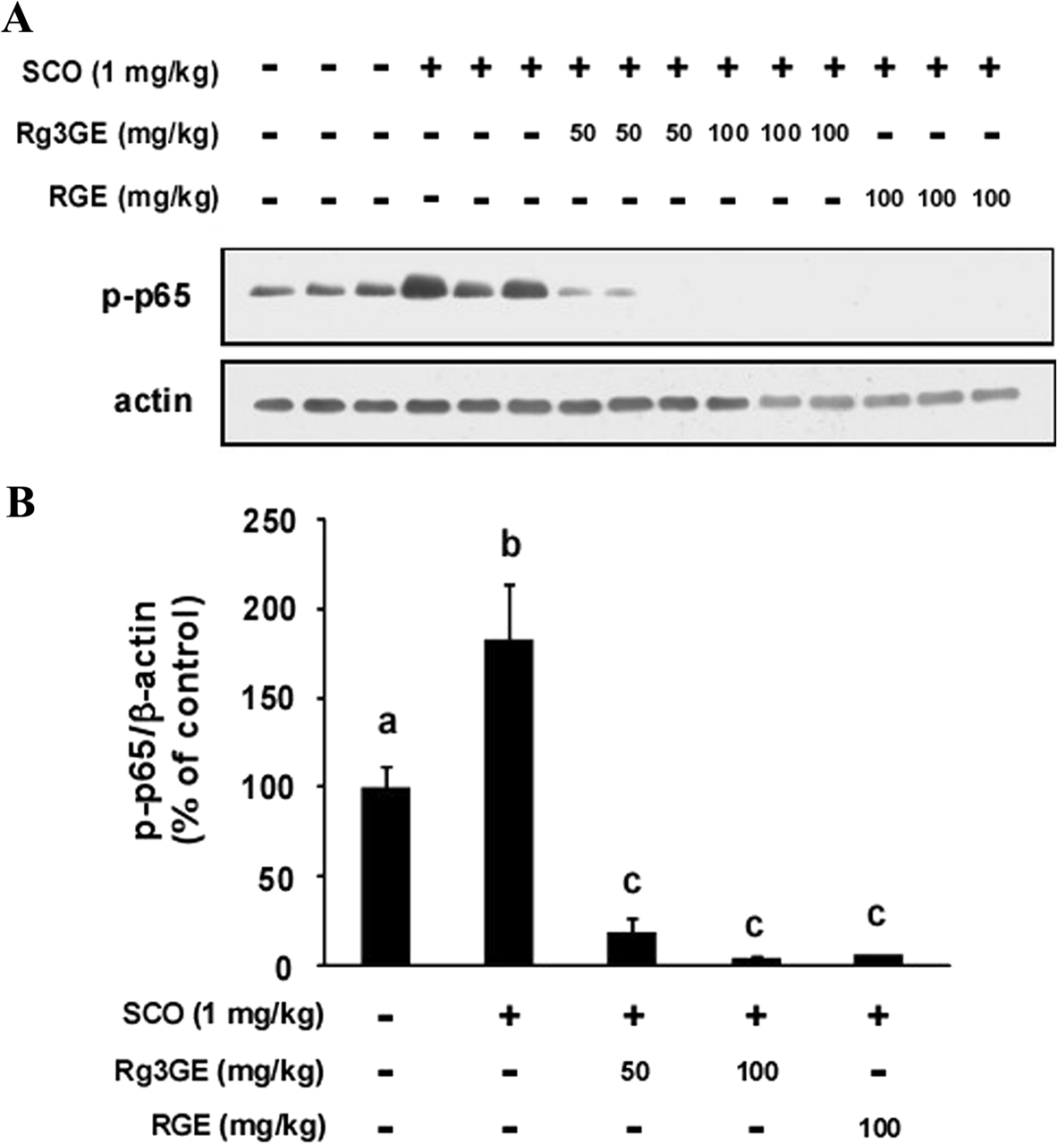
Effect of Rg3GE on scopolamine-induced NF-κB activation. **a** Levels of phosphorylated p65 were determined by Western blot analysis. β-actin was used as the loading control. **b** Quantitation of protein levels was achieved through densitometry analysis. Statistical analysis was performed by one-way ANOVA followed by Tukey’s post-hoc test. Data are expressed as mean ± SEM (*n* = 3). Letters (a–c) within a graph are significantly different from each other at *p* < 0.05

To determine the effect of Rg3GE on inflammatory signaling molecules in the hippocampus, we evaluated the phosphorylation of p65 by Western blot analysis (Fig. 6). We observed phosphorylation of p65 in the hippocampus of the non-induction + vehicle group. Treatment with scopolamine + vehicle significantly increased the levels of p-p65 in the hippocampus compared with non-induction + vehicle treatment. However, Rg3GE (50 and 100 mg/kg) administration suppressed the scopolamine-induced phosphorylation of p65, and showed even greater suppression of p65 phosphorylation compared to the non-induction + vehicle group. RGE (100 mg/kg) administration also inhibited the scopolamine-induced phosphorylation of p65 and showed even greater suppression of p65 phosphorylation compared to the non-induction + vehicle group. This observation suggests that Rg3GE has strong inhibitory activity on NF-κB activation and suppresses scopolamine-induced NF-κB activation.

## Discussion

Since ginsenoside Rg3 is not naturally present in *Panax ginseng* [13] and only occurs in red ginseng by way of a thermal process [15], we developed the method to increase the level of Rg3 without thermal processing of ginseng roots. The prepared Rg3-enriched ginseng extract (Rg3GE) contains 14.34 ± 0.28 mg/g of Rg3, and we investigated whether Rg3GE can ameliorate scopolamine-induced impairment of hippocampus-dependent spatial memory. We found that oral administration of Rg3GE significantly improved the search error of scopolamine-treated mice in the Morris water maze task, suggesting that Rg3GE is beneficial in the acquisition of spatial memory. Conversely, we observed that Rg3GE did not improve the retention of spatial memory retrieval in the probe test.

Chen et al. reported that ginsenoside Rg3 significantly decreases the levels of amyloid-β40 and amyloid-β42 in cultured cells and transgenic mice (Tg2576) that express high levels of amyloid-β [28]. Ginsenoside Rg3 protects cultured cortical neurons from glutamate-induced neurotoxicity [6]. In addition, it exhibits neuroprotective effects after permanent middle cerebral artery occlusion in rats by reducing lipid peroxides, scavenging free radicals, and improving energy metabolism [4]. Ginsenoside Rg3 increases ischemia-induced cell proliferation and survival in the dentate gyrus of adult gerbils [4, 29]. These observations potentiate the idea that Rg3 might be a promising compound that protects the brain and prevents neuronal disorders.

The action of acetylcholine is dependent upon acetylcholinesterase, which hydrolyzes acetylcholine [12, 30]. Inhibition of acetylcholinesterase activity has been the therapeutic approach used for dementia [31]. To elucidate the mechanisms underlying the neuropharmacological actions of Rg3GE, we assessed its effect on acetylcholinesterase activity in scopolamine-induced learning- and memory-deficient mouse brains. Rg3GE significantly inhibited the scopolamine-induced increase in acetylcholinesterase activity in the hippocampus. This suggests that Rg3GE may have an ameliorating effect on cholinergic dysfunction-induced memory impairment by enhancing cholinergic transmission. The beneficial effect of Rg3GE on the acquisition of spatial memory in scopolamine-treated mice is likely due to the prevention of scopolamine-induced dysregulation of acetylcholinesterase activity and acetylcholine level.

It has been reported that muscarinic acetylcholine receptors in the central nervous system inhibit systemic inflammation and the activation of muscarinic cholinergic transmission in the central nervous system lowers serum tumor necrosis factor (TNF) levels [32]. Since scopolamine is a nonselective muscarinic receptor antagonist, blockage of the muscarinic receptor by scopolamine might promote inflammation and TNF. Previous studies have confirmed that scopolamine not only impairs the cholinergic system, but also induces the expression of pro-inflammatory mediators and neurotoxic cytokines, such as cyclooxygenase-1 (COX-1), COX-2, TNF-α, and interleukin-1β (IL-1β) [23, 33–38]. It was also found that scopolamine modulates the stimulation of NF-κB pathways (i.e., the phosphorylation of IκBα and p65) [23, 33, 35]. Inflammatory reactions are related to the pathogenesis of neurodegeneration and cognitive impairment [39]. Chronic treatment with non-steroidal anti-inflammatory drugs (NSAIDs) reversed the cognitive deficits in scopolamine-treated mice [34]. Thus, scopolamine might cause learning and memory deficits through cholinergic neuronal injury complicated by NF-κB-mediated inflammation. Rg3GE might ameliorate the scopolamine-induced impairment of spatial memory by protecting the cholinergic system via inhibition of NF-κB signaling.

## Conclusions

We have developed a method to increase the level of Rg3 in ginseng roots without the need for a thermal process. The neuropharmacological effects of Rg3GE were tested. Rg3GE exhibited potent memory-stabilizing effects, as assessed by the Morris water maze test, and inhibited scopolamine-induced acetylcholinesterase activity in the hippocampus. Rg3GE also decreased the activation of NF-κB. The effects of Rg3GE were better than or similar to those of RGE. Our findings suggest that Rg3GE may improve cognitive deficits via the inhibition of acetylcholinesterase activity and inflammation.
